# Hydro-ethanolic extract of *Khaya grandifoliola* attenuates heavy metals-induced hepato-renal injury in rats by reducing oxidative stress and metals-bioaccumulation

**DOI:** 10.1016/j.heliyon.2022.e11685

**Published:** 2022-11-18

**Authors:** Arnaud Fondjo Kouam, Micheline Masso, Ferdinand Elombo Kouoh, Rodrigue Fifen, Ibrahim Njingou, Angèle Nkouatchoua Tchana, Frédéric Nico Njayou, Paul Fewou Moundipa

**Affiliations:** a Medical Research and Applied Biochemistry Laboratory, Department of Biomedical Sciences, Faculty of Health Sciences, University of Buea, P. O. Box 63, Buea, Cameroon; b Laboratory of Molecular Pharmacology and Toxicology, Department of Biochemistry, Faculty of Science, University of Yaoundé 1, P. O. Box 812, Yaoundé, Cameroon; c Laboratory of Animal Physiology, Department of Animal Biology and Physiology, Faculty of Science, University of Yaoundé 1, P. O. Box 812, Yaoundé, Cameroon

**Keywords:** Heavy metals, Hepato-renal toxicity, Oxidative stress, Metals-bioaccumulation, *Khaya grandifoliola*

## Abstract

People living in developing countries are exposed to hepato-renal injuries induced by heavy metals like lead (Pb), cadmium (Cd), and mercury (Hg) since drinking water supplied is often polluted with a high concentration of those metals. Accordingly, it is necessary to search for antidotes against heavy metals poisoning. Hence, medicinal plants bearing anti-hepatotoxic properties represent a credible option; and such plant is *Khaya grandifoliola*. However, there is a paucity of knowledge regarding its protective effect on heavy metals-induced hepato-renal toxicity. Thus, this study was designed to assess the protective effect of the hydro-ethanolic stem bark extract of *K. grandifoliola* (HKG) against hepato-renal injuries induced by chronic consumption of drinking water containing high contents of Pb, Cd, and Hg; in addition to the investigation of the chemical antioxidant properties of HKG. For the antioxidant assays, HKG was tested as a potential inhibitor of lipid peroxidation, reducer of ferric and phosphomolybdenum, and scavenger of hydroxyl and *2,2-*Diphenyl-Picryl-Hydrazyl radicals. Its protective effects were evaluated by daily co-treating rats with heavy metals solution (10 mL/kg b.w) containing 0.9, 0.58, and 1.13 ppm respectively for Pb, Cd and Hg and HKG (25 or 100 mg/kg b.w) for five consecutive months; and biochemical parameters associated to liver and kidneys functions, oxidative stress and metals bioaccumulation were assessed. HKG displayed a strong antioxidant capacity (IC50/EC50 range 3.95–17.17 μg/mL) correlated to its polyphenols content and comparable to that of Ascorbic acid. Serum levels of alkaline phosphatase, alanine/aspartate aminotransferase, and creatinine; renal and hepatic content of Cd and Pb, malondialdehyde and glutathione, activities of superoxide dismutase and catalase showed the protective effect of HKG, further evidenced by histopathological analysis. Taking together, these results demonstrated that HKG alleviates heavy metals-induced hepato-renal injuries in rats by reducing oxidative stress and metals-bioaccumulation.

## 1 Introduction

In his environment, humans are daily exposed to deleterious substances such as heavy metals whose toxicity has proven to be a major public health problem (Jaishankar et al., 2014). Heavy metals are considered as metals with density greater than 5 g/cm^3^. Among them, some as copper, zinc and iron are essential to maintain biochemical and physiological functions since in very low concentration they act as cofactors for several enzymes and proteins structures stabilizers (Järup, 2003; Su et al., 2017; Wang et al., 2020). However, other metals like mercury (Hg), cadmium (Cd), and lead (Pb), even at very low concentrations, can displace essential metals, therefore inhibiting enzyme activities or disrupting proteins structures, and consequently inducing deleterious effects to human health. This toxicity is exacerbated in some vital organs such as the liver and kidney, due to their bioaccumulation capability (Bortey-Sam et al., 2015).

Numerous industrial and mining activities represent the major sources of human exposure to heavy metals. Then these metals enter the body through different routes of contamination among which dermal route, inhalation of polluted air, ingestion of contaminated foods, and consumption of sullied water (Rehman et al., 2018). The latter route of transmission appears to be one of the prominent routes of contamination, especially in developing countries where the quality of drinking water is often associated with public health problems. Indeed, a study conducted to assess health risks to multiple heavy metals in drinking water in the city of Yaoundé-Cameroon, revealed a very high content of Pb, Cd, and Hg. The average level of each of these metals, which exceeded its corresponding European Commission Legislative Limits, 0.01, 0.003, and 0.001 part per million (ppm) respectively for Pb, Cd, and Hg, were 0.9, 0.58, and 1.13 ppm respectively (Angèle N. Tchana et al., 2018). Thus, regular consumption of such drinking water may constitute a risk to the health of the population.

The toxicity of heavy metals has been well described in the literature. Several studies suggest that one of the major mechanisms behind their toxicity is associated with the induction of oxidative stress, characterized by an overproduction of free radical and reactive oxygen species (ROS), which lead to the inactivation of both non-enzymatic and enzymatic antioxidant defense systems. In addition, the generated ROS can further bind to biological macromolecules and induce DNA damage, oxidation of protein and lipid membrane, leading to the disruption of cell membrane integrity and cell death (Balali-Mood et al., 2021; Rusyniak et al., 2010).

Due to the continuous exposure of humans to heavy metals and the health risk incurred, it is necessary to search for molecules or substances that can prevent or alleviate their harmful effects. Indeed, metal chelators have been used as a standard treatment for heavy metals poisoning, where the chelating agent binds to the metal ions and forms a complex, named chelates, to improve their elimination from the body. Unfortunately, metal chelation therapy has been linked with certain drawbacks, such as their redistribution from other organs to the brain thereby enhancing their neurotoxicity, as well as some serious adverse effects like hepatotoxicity and nephrotoxicity (Flora and Pachauri, 2010; Mehta and Flora, 2001). Accordingly, to prevent harmful effects of heavy metals on vital organs such as kidneys and liver, the use of natural antidotes, especially medicinal plants bearing anti-hepatotoxic properties, may represent a promising alternative. An example of such plants is *Khaya grandifoliola*, known for its use in indigenous medicine for the treatment of liver-related diseases, malaria, arthritis, anemia, and fever (Njayou et al., 2016; Olowokudejo et al., 2008). Pharmacological investigations have shown antimalarial, antibacterial, anti-inflammatory, and antifungal activities (Bickii et al., 2000; Mukaila et al., 2021). In addition, its ability to prevent toxin or drug-induced liver injury and inhibit hepatitis C virus (HCV) infection has been reported, thereby demonstrating its hepatoprotective properties (Galani et al., 2016; Njayou et al., 2015, 2016). Moreover, limonoids isolated from the *K. grandifoliola* as active ingredients were not only found to protect normal human hepatocytes cell line against cisplatin and acetaminophen-induced hepatotoxicity through activation of the anti-oxidative defense system but also to inhibit HCV infection by mainly targeting entry and replication steps of HCV life cycle (Arnaud et al., 2021; Fondjo Kouam et al., 2019; Kouam et al., 2017). However, there is a paucity of knowledge about its protective effect on heavy metals-induced hepato-renal toxicity. Accordingly, using rats as an animal model, this study aimed at assessing the potential toxicity of chronic consumption of drinking water containing high contents of Pb, Cd, and Hg similar to that found by Angèle N. Tchana et al. (2018) on the liver and kidneys; and concomitantly evaluating the protective effect of hydro-ethanolic stem bark extract of *K. grandifoliola* (HKG) on this multi-heavy metals induced hepatotoxicity and nephrotoxicity. Besides, the chemical antioxidant properties of HKG were also investigated.

## 2 Materials and methods

### 2.1 Reagents

Lead acetate (Pb(C_2_H_3_O_2_)_2_), Cadmium acetate (Cd(OCOCH_3_)_2_), mercury chloride (HgCl_2_), Silymarin, Ascorbic acid (Vitamin C), Gallic acid and Quercetin were all purchased from Sigma-Aldrich (St Louis, USA). The others reagents were of analytic grade.

### 2.2 Collection of plant sample and preparation of hydro-ethanolic extract of *K. grandifoliola* (HKG)

Stem bark of *K. grandifoliola* was collected in July 2018 in Foumban, Noun Sub-Division, West-Region, Cameroon). http://www.theplantlist.org was used to check the plant and the botanical identification was done at the Cameroon National Herbarium, where voucher specimen is kept under the reference number 23434 YA*.*

Hydro-ethanolic extract of *K. grandifoliola* was prepared as follows. The harvested plant sample was washed using tap water, air-dried, and ground. From the powder obtained, 500g were extracted at room temperature (25 °C) with 2 L of the solvent system ethanol-water (65:35, v/v) for 48 h with constant shacking. Whatman N°1 filter paper was then used to filter the mixture and the residue was re-extracted twice with the same volume of solvent. The collected filtrates were pooled and concentrated under reduced pressure with a rotary evaporator to remove ethanol. The remaining extract was dried in a lab dried oven at 40 °C to yield 77 g of HKG.

### 2.3 Phytochemical screening and high performance liquid chromatography analysis of HKA

The hydro-ethanolic extract of *K. grandifoliola* was subjected to qualitative analysis to detect the presence of major class of secondary metabolites such as phenolic compounds, flavonoids, Alkaloids, saponins, triterpenes, glycosides and tannins using standard method (Wadood, 2013). Quantitative analysis to determine polyphenol and flavonoid contents was also performed. The procedure used has been described elsewhere (Kouam et al., 2020). In brief, to quantify polyphenols content, 50 μL HKG (1 mg/mL in methanol) and 2.4 mL distilled water were mixed with 200 μL of Folin–Ciocalteu's reagent (1/10) and allowed to stand at 25 °C. After 5min, 200 μL of Na_2_CO_3_ 20 % were added. After 60min of incubation at 25 °C, the absorbance of the solution was recorded at 765 nm. A calibration curve using Gallic acid was established and used to determine the total polyphenols content which was expressed as milligrams of Gallic acid equivalent (GAE) per gram of extract.

Regarding flavonoids content, 25 μL of HKG dissolved at 1 mg/mL in methanol were mixed with 4.975 mL of aluminum tri-chloride (AlCl_3_) 2% and incubated for 30 min in the dark, the absorbance of the resulted yellowish solution was recorded at 420 nm. Using Quercetin as standard, a calibration curve was established and used to express flavonoid content as milligrams of Quercetin equivalent (QE) per gram of extract.

In addition, HPLC profile of HKG was analyzed as previously described (Kouam et al., 2017). Briefly, a column (Eclipse XDB-C8 column, 9.4 mm × 250 mm, 5 μm particle size) and a Liquid Chromatograph (Series 1200, Agilent Technologies, Ca, USA) equipped with a vacuum degasser, a quaternary pump, an autosampler and a Diode-Array-Detector (DAD) connected to Agilent ChemStation software were used. The mobile phase consisted of water (pump A) and acetonitrile (pump B). The working temperature and the injection volume were 28 °C and 5 μL respectively. Throughout the analysis, the flow rate was maintained at 1 mL/min and the elution conditions were as follows: B, 0–15 min, increasing gradient from 0 to 30 % B; 15–20 min, linear gradient 100 % B; 20–25 min, linear gradient 30 % B. 254 nm was used as detection wavelength.

### 2.4 Assessment of antioxidant activities of HKG

Chemical antioxidant activities of HKG were evaluated through the following assays: *in vitro* inhibition of lipid peroxidation; hydroxyl (HO°) and 2,2-Diphenyl-Picryl-Hydrazyl (DPPH) free radical scavenging assays; and ferric and phosphomolydenum reducing antioxidant power, where HKG and ascorbic acid (ASC) used as reference antioxidant were tested at the final concentrations of 0.01; 0.1; 1; 10 and 100 μg/mL.

#### 2.4.1 *In vitro* inhibition assay of lipid peroxidation induced in rat liver homogenate

The procedure described by Njayou et al. (2015) was used. In brief, lipid peroxidation was induced in rat liver homogenate with FeCl_2_–H_2_O_2_. Fifty μL of the tested sample (HKG or ASC) was mixed with 1 mL of 10% (w/v) rat liver homogenate; then, 50 μL of H_2_O_2_ (0.5 mM) and FeCl_2_ (0.5 mM) each, were added. The mixture was incubated for 60 min at 37 °C before the addition of 1 mL of trichloroacetic acid (15%, w/v) and thiobarbituric acid (0.67%) respectively. The resulted mixture was heated for 15 min at 100 °C in a water bath, followed by centrifugation (3000 g, 5 min, 4 °C). Finally, the supernatant was collected and the absorbance recorded at 532 nm. The result expressed as percentage of inhibition was determined with formula (1).

(1) Inhibition of lipid peroxidation (%) = 100 × ([A_0_ – A_1_]/A_0_)

where A_0_ represents the absorbance of the control and A_1_ represents the absorbance of the tested sample.

#### 2.4.2 Hydroxyl radical scavenging assay

The procedure based on Fenton reaction through the generation of hydroxyl radical *in vitro* was used (Su et al., 2009). In brief, in each test tube was successively added 444 μL of FeSO4 (3 mM), 635 μL of H2O2 (1 mM), 635 μL of distilled water, 32 μL of plant sample or standard to achieve the desired concentration, and 254 μL of Sodium Salicylate (10 mM). The mixture was then incubated for 60 min at 37 °C and the absorbance read at 532 nm. The percentage of hydroxyl radical scavenging was determined using formula (1).

#### 2.4.3 DPPH radical scavenging assay

DPPH radical scavenging was determined as described elsewhere (Kouam et al., 2020). Briefly, 3.1 mL of DPPH solution (40 μg/mL in methanol) was mixed with 50 μL of the tested sample. The absorbance was read 30 min later after incubation at 25 °C in the dark. The percentage of scavenging activity of DPPH radical was also determined using formula (1).

#### 2.4.4 Ferric Reducing Antioxidant Power (FRAP) assay

FRAP assay was performed as outlined by (Chuisseu et al., 2020) with slight modification. The FRAP mixture consisted of 2.2 mL phosphate buffer (0.2 M; pH 6.6) and 2 mL potassium ferrocyanate (0.25%, w/v). Then, 100 μL of HKG or ASC was added and the mixture incubated at 50 °C for 20 min. Then, 2 mL of trichloroacetic acid (10%, w/v) was added and the resulted mixture was centrifuged at 3000 g for 10 min. 2 mL of supernatant were mixed with 500 μL of freshly prepared FeCl3 (0.02%, w/v) and allowed to stand up for 10 min at 25 °C before recording the absorbance at 700 nm against the blank where the tested sample was replaced by distilled water. The percentage of reducing ability was calculated using formula (2).

(2) Reducing ability (%) = 100 × ([A_1_ – A_0_]/A_1_)

where A_1_ is the absorbance of the tested sample while A_0_ is the absorbance of control.

#### 2.4.5 Phosphomolybdenum reducing assay (total antioxidant capacity)

Total antioxidant capacity of HKG was evaluated using the phosphomolybdenum assay as reported previously (Njayou et al., 2015). In test tube were successively added 300 μL of HKG or ascorbic acid, and 3 mL of reaction mixture containing 0.6 M sulphuric acid; 4 mM ammonium molybdate, and 28 mM sodium phosphate. The resulted mixture was heated at 95 °C for 90 min. After cooling down, the absorbance of the solution was measured at 695 nm. For the control, distilled water was used instead of tested sample. The percentage of reduction was then determined using formula (2).

#### 2.4.6 Determination of half efficient/inhibitory (EC50/IC50) and correlations between total polyphenols content and the antioxidant activities

For each antioxidant assay, the graph “Antioxidant Activity vs. Log [HKG or ASC]” was plotted using GraphPad Prism 5.03 software and the corresponding EC_50_ or IC_50_ value was determined. Then, linear regression analysis was used to determine the correlation between the corresponding antioxidant activity and polyphenols content of HKG. Accordingly, total polyphenols was quantified in four different solutions (0.1, 1, 10, and 100 μg/mL). Then, for each chemical antioxidant assay, the activity of HKG at each tested concentration was plotted against total polyphenol content using Microsoft Excel 2013, and the correlation coefficient (r^2^) value was deduced from the graph.

### 2.5 Assessment of the protective effect of HKG on multi-heavy metals-induced hepato-nephrotoxicity

#### 2.5.1 Animal and experimental design

Male healthy *Wistar* albino rats, weighing between 80–100 g were used. They were provided by the Animal House of the Laboratory of Pharmacology and Toxicology (University of Yaoundé 1). They were maintained in a plastic cage with access to a standard diet composed of ground whole corn, ground wheat bran, ground whole soybean, dried skim milk, fish meal, soybean oil, salt, vitamin and mineral mix; and tap water ad libitum, under standard laboratory conditions of temperature (about 27 ± 3°) and 12 h day light-dark cycle. All the procedures were in respect to the ARRIVE guidelines on animal care and approved by the Institutional Joint Review Board for Animals and Humans Bioethics of the University of Yaounde I-Cameroon (Ethical approval No. 2019/17-05/SG/IJRBAHB/UYI).

The solution of heavy metals mixture was prepared in demineralized water at the concentrations of 0.9, 0.58 and 1.13 ppm respectively for Pb, Cd and Hg as found by Angèle N. Tchana et al. (2018). HKG, and silymarin, used as hepatoprotective reference agent were prepared in 1% carboxyl-methylcellulose (CMC). Both solution of heavy metal mixture and plant extract or silymarin were administered by oral route to the animals at the dose of 10 mL/kg body weight (b.w) with 12 h interval between administration of heavy metals mixture and plant extract. A total of 36 animals were included in the study. After 7 days of acclimation, they were divided into 6 groups of 6 animals each and treated daily for 5 consecutive months as follows:

Group I received demineralized water and 1% CMC and served as negative control group.

Group II received heavy metals mixture and 1% CMC and served as induced toxicity group.

Group III, Group IV and Group V served as induced toxicity and treated groups. They all received heavy metals mixture and treated either with silymarin at the dose of 100 mg/kg b.w (Group III), or HKG at the dose of 25 mg/kg b.w (Group IV) or 100 mg/kg b.w (Group V).

Group VI received demineralized water and HKG at the dose of 100 mg/kg b.w, and served as positive control group.

For Group I and Group II, at the end of each month, blood sample was collected by orbital puncture to monitor the occurrence of liver and kidney injury induced by the administration of heavy metals mixture. At the end of treatment period, animals were sacrificed under anesthesia 24 h after the last administration of HKG. Then, blood samples, kidneys and liver tissues were collected for biochemical and histopathological analysis.

#### 2.5.2 Evaluation of liver and kidneys function markers

Kidney and liver damage induced by heavy metals mixture were assessed through measurement of serum content of creatinine, and serum activities of alkaline phosphatase (PAL), alanine aminotransferase (ALT) and aspartate aminotransferase (AST) respectively. Serum was obtained by centrifugation (3000 g, 15 min, 4 °C) of blood samples. Serum activities level of ALT and AST were then determined as previously described by Reitman and Frankel (1957). Serum creatinine content and alkaline phosphatase activity were determined using commercial kits (Cat N° REF_92314 and Cat N° REF_80107 for alkaline phosphatase and creatinine kits respectively) purchased from BioLabo (Les hautes Rives 02160, Maizy, France). The procedures were performed according to the manufacturer's instructions.

#### 2.5.3 Evaluation of oxidative stress markers

Oxidant status of liver and kidneys tissues was assess in one hand by determining liver and kidneys content of malondialdehyde (MDA), and end product of lipid oxidation; and in other hand, by assessing enzymatic and non-enzymatic antioxidant defense systems through measurement of superoxide dismutase (SOD) and catalase (CAT) activities, and reduced glutathione (GSH) content respectively. For these assays, 10% liver or kidney tissue homogenate was prepared in Tris-HCl 20 mM, KCl 150 mM, pH 7.4 buffer supplemented with 0.2% Halt Protease Inhibitor Cocktail EDTA-Free (Thermo Fisher Scientific), and used.

##### 2.5.3.1 Assessment of lipid peroxidation

Lipid peroxidation, in terms of thiobarbituric acid reactive substances (TBARS) formation, was determined as previously described (Kouam et al., 2017) with slide modification. Briefly, 0.5 mL of 10% tissue homogenate was mixed with 1 mL TCA (20%, v/v) and 1 mL TBA (0.67%, v/v), and heated for 20 min at 100 °C. After cooling, the precipitate was removed by centrifugation (3000 g, 15 min, 4 °C) and the absorbance of the supernatant was recorded at 535 nm against a blank containing all the reagents except 10% tissue homogenate, replaced by 0.5 mL Tris-HCl buffer. The TBARS content was estimated in terms of malondialdehyde (MDA) and calculated using the extinction coefficient of MDA, which is 1.56 × 10^5^ M^−1^.Cm^−1^.

##### 2.5.3.2 Assessment of superoxide dismutase (SOD) activity

The activity of SOD in 10% tissue homogenate was measured as reported previously (Ghosh et al., 2008). The reaction mixture included 1.2 mL sodium pyrophosphate buffer (50 mM; pH 8.3), 100 μL phenazine methosulfate (186 μM), 300 μL nitroblue tetrazolium (300 μM), 200 μL NADH (720 μM), the adjusted volume of 10% liver or kidney homogenate containing 10 μg of protein (protein concentration in tissue homogenate was measured using Lowry's reagent) and distilled water in a total volume of 3 mL. The assay was initiated by addition of NADH. After incubation at 30 °C during 90 s, 1 mL glacial acetic acid was added to stop the reaction; and the resulted mixture was stirred vigorously and shaken with 4 mL of n-butanol. The mixture was incubated for 10 min at 25 °C before centrifugation (3000 g, 5 min, 25 °C). Then, the optical density of the chromogen in the butanol layer was recorded at 560 nm. One unit of enzyme activity was defined as enzyme concentration required to inhibit absorbance of chromogen production by 50% per minute and SOD activity was expressed as specific activity in Unit/min/mg protein.

##### 2.5.3.3 Assessment of catalase (CAT) activity

CAT activity was evaluated according to the method described by Aebi (1984). In brief, 1 mL phosphate buffer (50 mM; pH 7.2) was added to 990 μL H_2_O_2_ (10 mM) solution and 10 μL of 10% liver or kidney homogenate was added. The decrease of the optical density of H_2_O_2_ was followed at 240 nm and recorded at 20 s and 80 s. The CAT activity was then calculated by using the following equation: CAT Activity (Unit/min/mg of protein) = (2.3033/ΔT) × (logA1/A2)/Q_protein_ where A1 is the optical density at 20 s; A2 is the optical density at 80 s; **ΔT** is the variation in time (1min) and Q_protein_ is the amount of protein (mg) 10% tissue homogenate.

##### 2.5.3.4 Determination of reduced glutathione (GSH) content

The procedure described by Ellman (1959) was used. 50 μL of 10% liver or kidney homogenate was mixed to 3 mL Ellman's reagent (0.05 mM DTNB in phosphate buffer 0.1 M pH 6.5) and at 25 °C for 60 min and the optical density was recorded at 412 nm. Then, GSH content was calculated using its molar extinction coefficient (**ε**_**GSH**_ = 13,600 M^−1^.Cm^−1^).

#### 2.5.4 Assessment of Pb and Cd bioaccumulation in the liver and kidneys

Accumulation of Pb and Cd within the liver and kidneys was performed as described by Angèle N. Tchana et al. (2018) with slight modifications. In the procedure, all glassware and tubes were washed in 10% nitric acid solution and rinsed with demineralized water prior use, in order to minimize further metal contamination of sample. A wet digestion of each organ was separately conducted. One g of liver or kidney was introduced in a flask, then 10 mL of digestion solution (75% nitric acid/36% hydrochloric acid, 1:3; v/v) were added. The flask was kept in a reflux device and heated at 90 °C for about 2–3 h till the solution become clear. After cooling down, the mineralized sample was transferred into a measuring flask using Whatman filter paper. The volume of filtrate obtained was made up to 50 mL with demineralized water and stored at 4 °C until use. Samples were mineralized in duplicate, and metal contents were determined using an Atomic Absorption Spectrophotometer (Burk Scientific, In., Fort Point., USA.). In brief, 25 mL of mineralized sample were introduced into the Air-Acetylene flame of the spectrophotometer, with a lamp current of 4 mA, in which vaporization and atomization took place. Wavelength of 217 nm and 288.8 nm were used for the assessment of Pb and Cd respectively. Standard solutions with known concentrations: 0.125; 0.25; 0.5; 1; 2.5; 5; and 10 ppm of the respective metal prepared with demineralized water were used for the calibration of the spectrophotometer and allowed to read the concentration (ppm) of metal.

#### 2.5.5 Histopathological examination

Histopathological examination was performed as previously described (Kouam et al., 2020). Briefly, a portion of kidney and liver tissues were fixed in 10% formalin solution. After dehydration, the tissues were embedded in paraffin. Then, a rotary microtome was used to obtain paraffin embedded fragment sections of 4–5 μm. The sections were mounted on glass slides and stained with hematoxylin and eosin for visualization of histological changes under optic microscope (×-100).

### 2.6 Statistical analysis

The results were expressed as mean ± standard deviation (SD) of three independent assays in triplicate (for animal study, n = 6). The differences between the mean values of different groups were analyzed by one-way analysis of variance (ANOVA) followed by Bonferroni's post-test using GraphPad Prism 5.03 statistical software package (Graph Pad Inc., USA). Differences between compared groups were considered significant for p < 0.05.

## 3 Results

### 3.1 Secondary metabolites, polyphenols, and flavonoid contents and HPLC fingerprint of HKG

**Figure 1:**
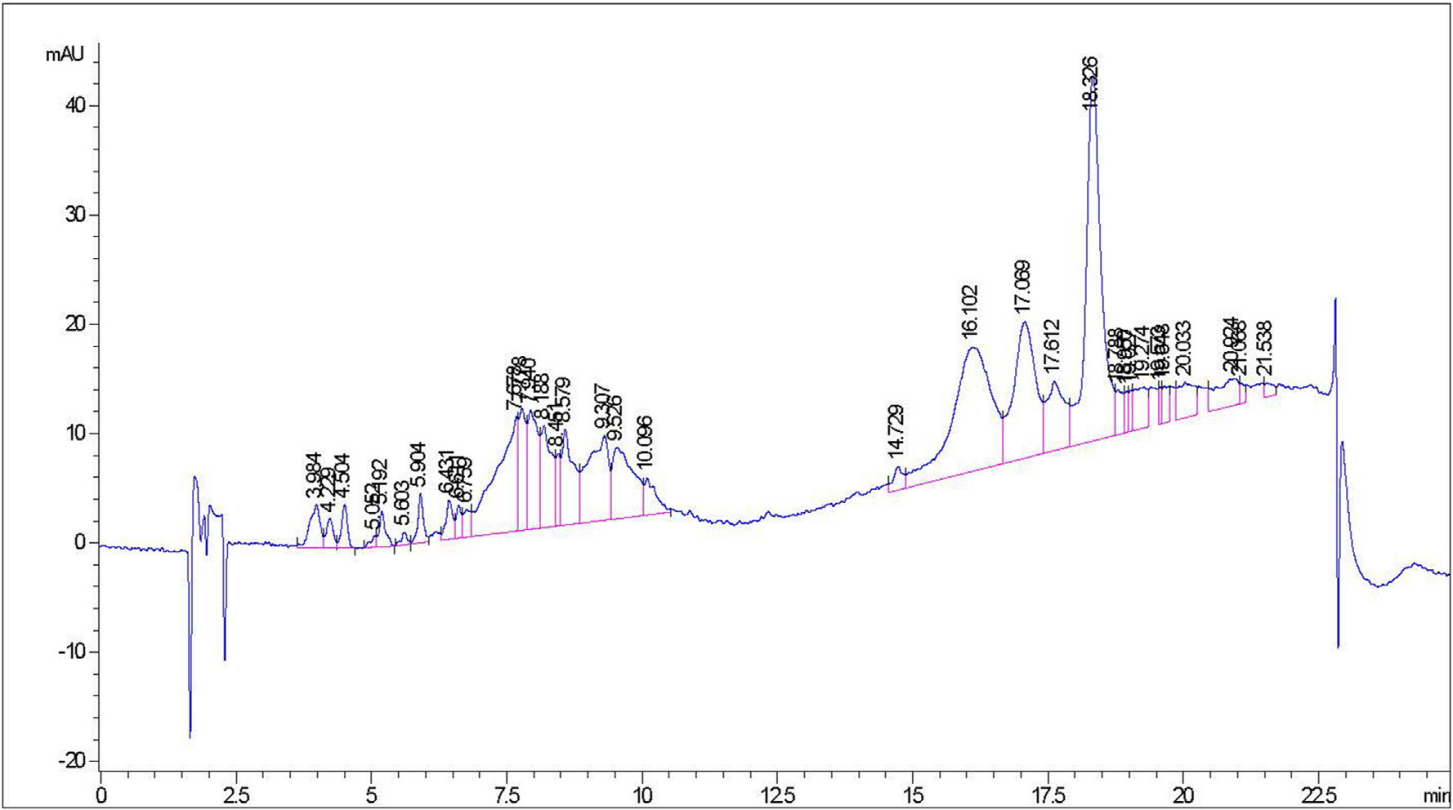
Chromatographic profile of HKG. After extraction, HKG was subjected to HPLC analysis through HPLC-ACN-Standard-Method. An Eclipse XDB-C8 column (9.4 × 250 mm, 5 μm particle size) was used; The mobile phase consisted of: (A) water and (B) acetonitrile; The elution condition were: B, 0–15 min, increasing gradient from 0 to 30 % B, 15–20 min, linear gradient 100 % B; 20–25 min, linear gradient 30 % B; flow rate: 1 mL/min; injection volume: 5 μL. HKG: Hydro-Ethanolic (35:65, v/v) extract of *K. grandifoliola.*

Qualitative and quantitative analyses were performed to assess the phytochemical profile of the hydro-ethanolic (35:65, v/v) extract of *K. grandifoliola* (HKG). The qualitative screening revealed that HKG contains phenolic compounds, flavonoids, saponins, alkaloids, triterpenes, glycosides, and tannins as the major class of secondary metabolites while quantitative analysis showed that the phenolic compounds and flavonoids content are 49.44 ± 2.32 mg GAE/g of extract and 18.54 ± 3.87 mg QE/g of extract respectively (Table 1). HPLC fingerprint of HKG revealed several peaks with various retention times detected at 254 nm, probably denoting the availability of aromatic compounds within the extract (Figure 1).

**Table 1:** Major class of secondary metabolites tested positive, Flavonoids and Total polyphenols content of HKG.

HKG	Major class of secondary metabolites detected	Flavonoids (mg QE/g of extract)	Total polyphenols (mg GAE/g of extract)
	Phenolic compounds, Flavonoids, Saponins, Alkaloids, Triterpenes, Glycosides and Tannins	18.54 ± 3.87	49.44 ± 2.32

HKG: Hydro-Ethanolic (35:65, v/v) extract of *K. grandifoliola*; mg GAE/g of extract: milligrams of Gallic acid equivalent per gram of extract; mg QE/g of extract: milligrams of Quercetin equivalent per gram of extract. Values are means ± SD of three independents experiments in triplicate.

### 3.2 HKG exhibits strong *in vitro* antioxidant activities

**Figure 2:**
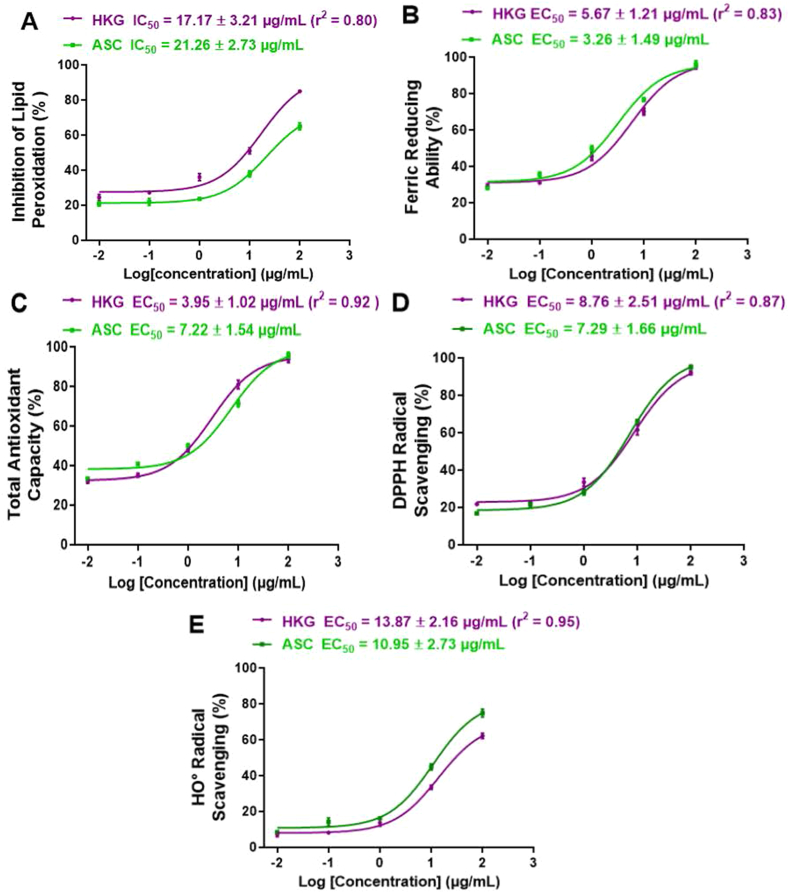
*In vitro* Antioxidant activities of HKG. (A): Inhibition of lipid peroxidation assay in rat liver homogenate; (B): Ferric Reducing Antioxidant Power (FRAP) assay; (C): Total antioxidant capacity assay; (D): 2,2-Diphenyl-Picryl-Hydrazyl (DPPH) free radical scavenging assays; (E): Hydroxyl radical scavenging assay; HKG: Hydro-Ethanolic (35:65, v/v) extract of *K. grandifoliola.* ASC: Ascorbic acid; EC_50_: Half efficient concentration; IC_50_: Half inhibitory concentration. R^2^: Correlation coefficient between the antioxidant activity and total polyphenol content. Values expressed as means ± SD of three independent experiments in triplicate.

The chemical antioxidant activities of HKG, along with that of Ascorbic acid (ASC) used as a reference antioxidant compound, are depicted in Figure 2. Overall, HKG and ASC showed concentration-dependent activities for the five end-point chemical antioxidant models assessed. On one hand, concerning Ferric reducing ability (Figure 2B), DPPH radical scavenging (Figure 2D), and Hydroxyl radical scavenging (Figure 2E) assays, HKG displayed lower antioxidant activities with EC_50_ of 5.67 ± 1.21; 8.76 ± 2.51 and 13.87 ± 2.16 μg/mL respectively, compared to that of ASC with EC_50_ of 3.26 ± 1.49; 7.29 ± 1.66 and 10.95 ± 2.73 μg/mL respectively. In contrast, regarding the Inhibition of lipid peroxidation (Figure 2A) and total antioxidant capacity (Figure 2E) assays, HKG exhibited strong antioxidant activities with IC_50_/EC_50_ of 17.17 ± 3.21 and 3.95 ± 1.02 μg/mL, respectively, than ASC with IC_50_/EC_50_ of 21.26 ± 2.73 and 7.22 ± 1.54 μg/mL respectively. Furthermore, a positive correlation was observed between the phenolic compounds content of HKG and the five cell-free system antioxidant studied with r^2^ (correlation coefficient) of 0.80; 0.83; 0.93; 0.87, and 0.95 respectively for the inhibition of lipid peroxidation (Figure 2A), Ferric reducing ability (Figure 2B), total antioxidant capacity (Figure 2C), hydroxyl radical scavenging (Figure 2D) and DPPH radical scavenging (Figure 2E) assays.

### 3.3 Effect of heavy metals mixture on the general conditions of rats

During the 5 months of treatment, neither death nor appearance signs of toxicity (salivation, reduction of locomotion, dizziness) were observed in different groups of experimental animals. Supplementary Figure S1 depicts the data on the body weight gain evolution. No significant (*P* > 0.05) difference in body weight changes was noted in treated and untreated animals during the first two months. However, a significant (*P* ˂ 0.05) in body weight gain was observed in heavy metals-intoxicated and non-treated rats from the third to the fifth month, as compared to non-intoxicated rats. However, daily co-treatment of rats with HKG (25 or 100 mg/kg b.w) or silymarin (100 mg/kg b.w) significantly (*P* ˂ 0.05) reduced heavy metals-induced weight loss. In contrast, administration of HKG (100 mg/kg b.w) alone did not significantly (*P* > 0.05) affect body weight evolution during the entire period of treatment.

### 3.4 Heavy metals mixture induced hepato-renal damage in rats

**Figure 3:**
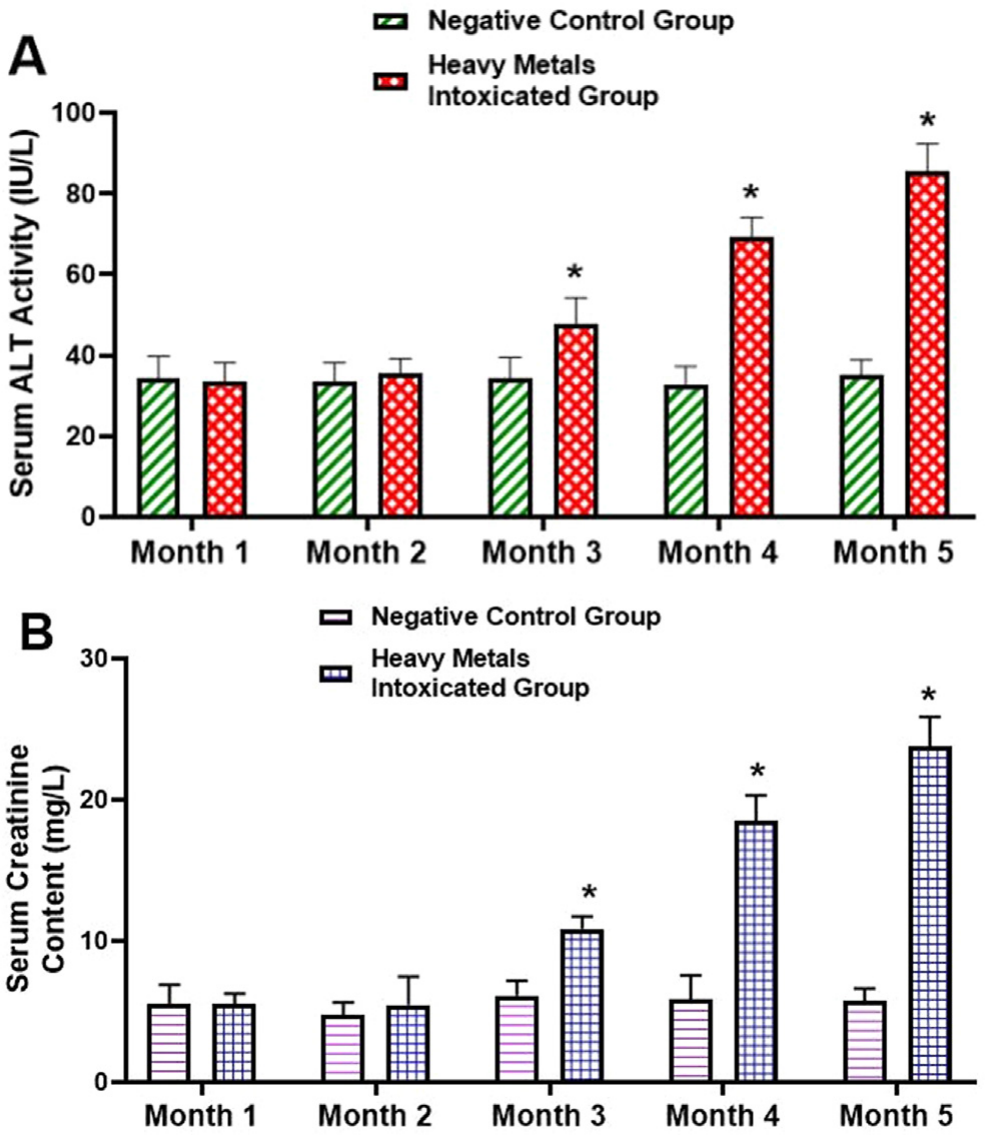
Time-dependent effect of heavy metals mixture on liver and kidneys function markers. Animals were treated daily with either demineralized water (negative control group) or heavy metal mixture (heavy metals intoxicated group) for five consecutive months. At the end of each month, blood sample was collected to assess liver and kidneys damage by measuring serum ALT activity and creatinine content, respectively. (A): Serum activity of ALT as marker of hepatic function. (B): Serum creatinine content as marker of kidneys function. Values are expressed as means ± SD, n = 6; ∗ values significantly different compared to negative control group (P ˂ 0.05). ALT: alanine aminotransferase.

The effects of chronic administration of Pb, Cd, and Hg at the respective concentration of 0.9; 0.58, and 1.13 ppm on liver and kidneys functions parameters in rats were monitored for five consecutive months through measurement of ALT activity and Creatinine content in the serum. As presented in Figure 3, during the first two months, administration of heavy metals mixture did not significantly (*P* > 0.05) alter ALT activity (Figure 3A) and Creatinine content (Figure 3B) in the serum, as compared to normal control rats. However, from the third till the fifth month, daily administration of heavy metals mixture induced hepatic and renal damage as evidenced by significant (*P* ˂ 0.05) and gradually increased activity of ALT (Figure 3A) and Creatinine content (Figure 3B).

### 3.5 HKG attenuates hepato-renal damages induced by chronic administration of the heavy metals mixture

The effect of chronic administration of heavy metals mixture and the protective effect of HKG on liver and kidney function biomarkers are presented in Table 2. Compared to normal control rats, serum activities of liver enzymes, ALT, AST and PAL, and renal product, Creatinine were significantly increased (*P* ˂ 0.05) in heavy metals intoxicated and non-treated rats. In contrast, daily co-treatment of rats with HKG at the dose of 25 mg/kg b.w or 100 mg/kg or silymarin (100 mg/kg) significantly (*P* ˂ 0.05) reduced the elevation of serum levels of ALT, AST, PAL, and Creatinine, when compared to heavy metals intoxicated and non-treated rats. However, these serum biochemical parameters remain unchanged in rats treated with HKG (100 mg/kg b.w) when compared to normal control rats.

**Table 2:** Kidneys and liver function markers in the serum of rats exposed to heavy metals mixture and treated with HKG.

Treatments	Kidney and liver serum markers			
	Creatinine (mg/dL)	ALP (IU/L)	ALT (IU/L)	AST (IU/L)
**Control**	7.52 ± 1.07	134.58 ± 3.98	32.47 ± 4.66	21.53 ± 3.07
**Heavy metals**	23.75 ± 2.10^**Δ**^	213.27 ± 4.37^**Δ**^	83.52 ± 4.89^**Δ**^	67.29 ± 5.84^**Δ**^
**Heavy metals + Sil (100 mg/kg)**	13.53 ± 2.87**∗**	163.56 ± 3.64**∗**	47.67 ± 4.21**∗**	31.28 ± 4.33**∗**
**Heavy metals + HKG (25 mg/kg)**	12.48 ± 2.09**∗**	173.40 ± 5.56**∗**	59.34 ± 5.73**∗**	37.04 ± 3.10**∗**
**Heavy metals + HKG (100 mg/kg)**	10.63 ± 0.87**∗**	155.27 ± 3.92**∗**	41.28 ± 3.95**∗**	26.77 ± 2.14**∗**
**HKG (100 mg/kg)**	8.31 ± 1.74^ns^	137.60 ± 4.23^ns^	35.48 ± 4.13^ns^	19.34 ± 3.85^ns^

Animals were treated daily with either demineralized water (negative control group), heavy metals mixture (heavy metals intoxicated group), HKG or co-treated with heavy metals mixture and HKG or silymarin for five consecutive months. At the end of the treatment, serums were prepared from blood samples and used to assess the effect of HKG on the biomarkers of kidneys (creatinine) and liver (ALP, ALT and AST) functions. Values are means ± SD, n = 6; ^Δ^ values significantly different when compared to control group (P ˂ 0.05); ^ns^ values non significantly different when compared to control group (P > 0.05); ∗ values significantly different compared to heavy metals-intoxicated group (P ˂ 0.05) using ANOVA followed by Bonferroni's post-test. ALP: Alkaline phosphatase; ALT: Alanine aminotransferase; AST: Asparte aminotransferase; Control: Negative control group; Heavy metals: Heavy metals-intoxicated group. Sil: Silymarin; HKG: Hydro-Ethanolic (35:65, v/v) extract of *K. grandifoliola*.

**Figure 4:**
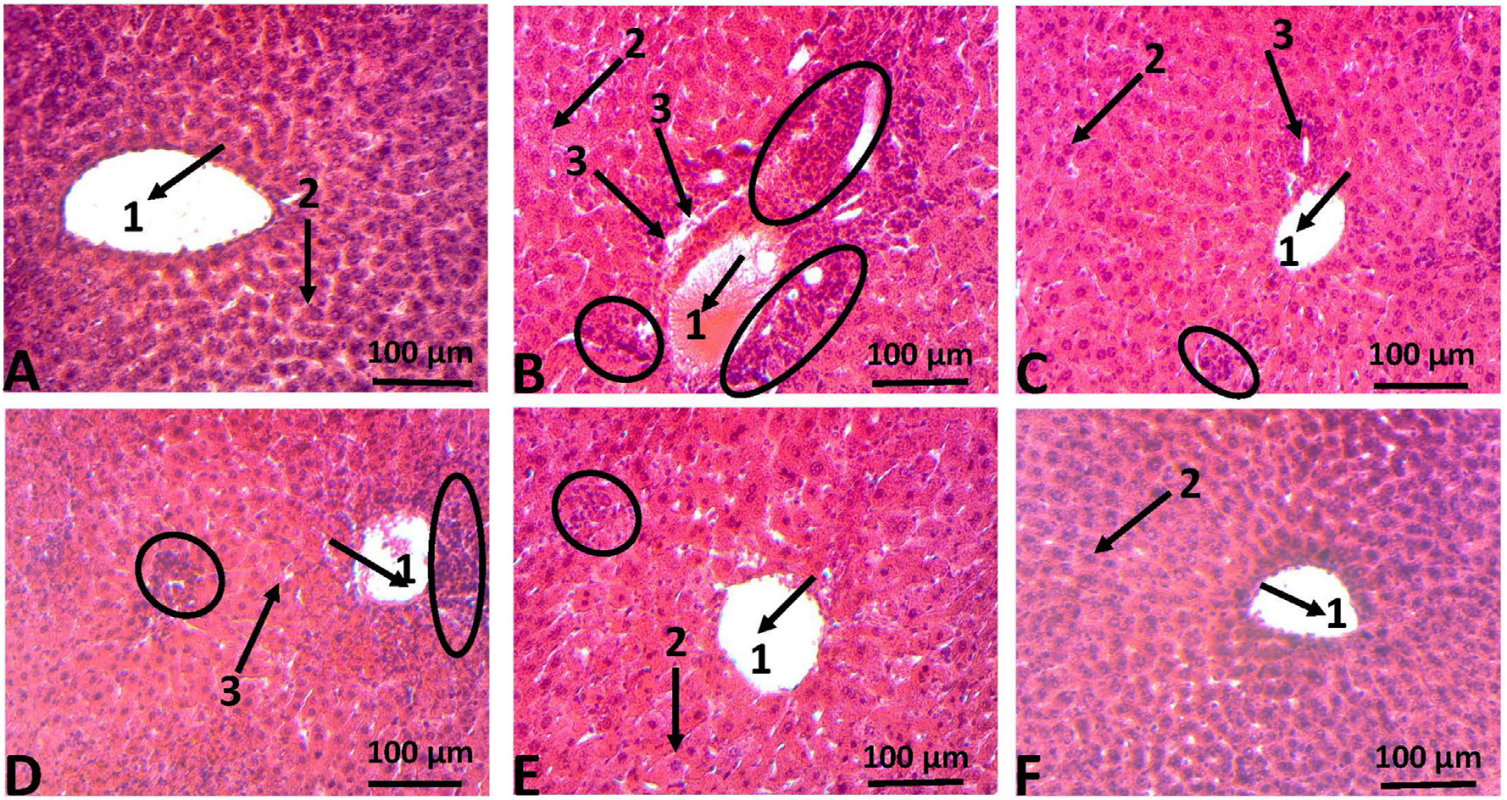
Microphotographs of rats' liver exposed to heavy metals mixture and treated with HKG. Animals were treated daily with either demineralized water (negative control group), heavy metals mixture (heavy metals intoxicated group), HKG or co-treated with heavy metals mixture and HKG or silymarin for five consecutive months. At the end of the treatment, the liver was removed, fixed and embedded in paraffin, and sections were stained with hematoxylin-eosin (H-E) and observed under light-microscope, magnificence ×-100. (A) Liver section of negative control rat showing normal hepatic architecture: centro-lobular vein (arrow 1) and normal hepatocyte (arrow 2). (B) Liver section of heavy metal-treated rat presenting massive inflammatory cell infiltration (circle) around centro-lobular vein (arrow 1) and severe hepatocytes necrosis (arrow 3). (C) Liver section of rat co-treated with heavy metals mixture + silymarin (100 mg/kg/b.w/day) presenting nearly normal hepatic architecture with a few hepatocyte necrosis (arrow 3) and moderate inflammatory cell infiltration. (D) Liver section of rat co-treated with heavy metals mixture + HKG (25 mg/kg/b.w/day) presenting mild hepatocytes necrosis (arrow 3) and mild inflammatory cell infiltration (circle). (E) Liver section of rat co-treated with heavy metals mixture + HKG (100 mg/kg/b.w/day) presenting almost normal hepatic architecture with absence of hepatocytes necrosis and very few inflammatory cells infiltration. (F) Liver section of rat treated with HKG (100 mg/kg/b.w/day) presenting normal hepatic architecture without hepatocytes necrosis and inflammatory cells infiltration.

**Figure 5:**
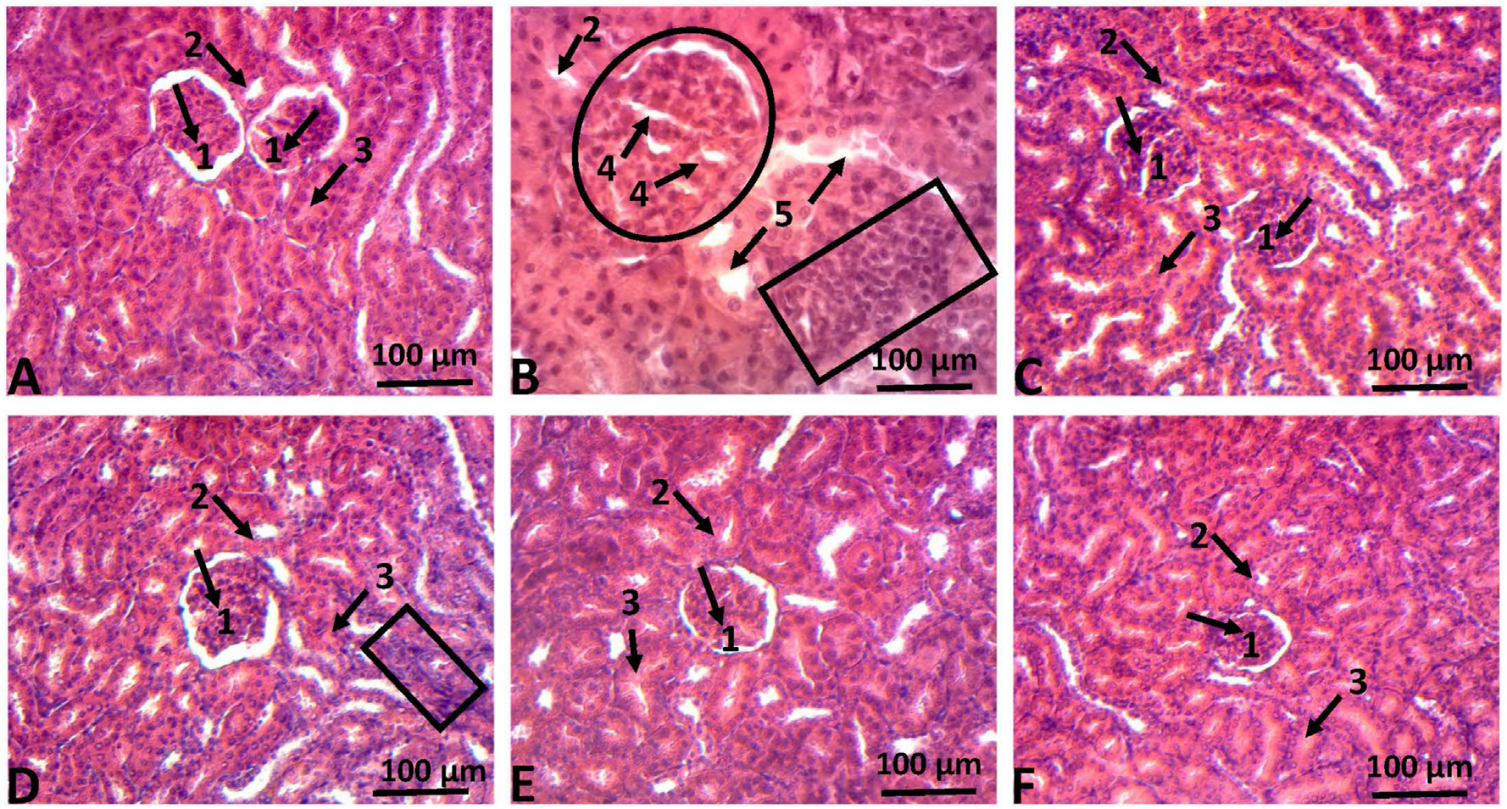
Microphotographs of rats' kidneys exposed to heavy metals mixture and treated with HKG. Animals were treated daily with either demineralized water (negative control group), heavy metals mixture (heavy metals intoxicated group), HKG or co-treated with heavy metals mixture and HKG or silymarin for five consecutive months. At the end of the treatment, the kidneys was removed, fixed and embedded in paraffin, and sections were stained with hematoxylin-eosin (H-E) and observed under light-microscope, magnificence ×-100. (A) Kidney section of negative control rat presenting normal kidney architecture with glomerulus (arrow 1), proximal tubules (arrow 2) and distal tubule (arrow 3). (B) Kidney section of heavy metal-treated rat presenting massive inflammatory cell infiltration (rectangle), glomerulus hypertrophy (circle) with nephron necrosis (arrow 4) and distal tubule necrosis (arrow 5). (C) Kidney section of rat co-treated with heavy metals mixture + silymarin (100 mg/kg, b.w) presenting nearly normal kidney architecture. (D) Kidney section of rat co-treated with heavy metals mixture + HKG (25 mg/kg, b.w) presenting almost normal kidney architecture with moderate inflammatory cell infiltration (rectangle). (E) Kidney section of rat co-treated with heavy metals mixture + HKG (100 mg/kg, b.w) presenting almost normal kidney architecture with absence of glomerulus hypertrophy and inflammatory cells infiltration. (F) Kidney section of rat treated with HKG (100 mg/kg, b.w) presenting normal kidney architecture without glomerulus hypertrophy, distal tubule necrosis and inflammatory cells infiltration.

In addition, hematoxylin-eosin staining was used to further analyze the protective effect of HKG on heavy metals-induced histological changes in rats' liver and kidneys. As shown in Figure 4, in both normal control rats and those treated only with 100 mg/kg b.w of HKG, normal hepatic histology was preserved (Figure 4A and Figure 4F) with the normal hepatic lobular architecture around the Centro-lobular vein. However, in liver tissues of heavy metals intoxicated rats, severe histopathological changes were observed, among which impairment of hepatic lobule architecture, severe hepatocytes necrosis, and massive inflammatory cells infiltration surrounded by the Centro-lobular vein (Figure 4B). On contrary, the normal liver structure was almost preserved in HKG or silymarin co-treated rats (Figure 4C and Figure 4E) except for a few hepatocytes necrosis and moderate lymphocytes infiltration observed in heavy metals +25 mg/kg b.w of HKG (Figure 4D). Likewise, in the control and HKG (100 mg/kg b.w) treated rat's kidney, the normal architecture of renal tissues was maintained, with unaltered glomerulus, proximal and distal tubules (Figure 5A and Figure 5F). On contrary, the kidney section of heavy metals intoxicated rats (Figure 5B) showed massive infiltration of inflammatory cells, glomerulus hypertrophy, nephron and distal tubules necrosis. However, daily co-treatment of intoxicated rats with HKG (25 or 100 mg/kg b.w) or silymarin (100 mg/kg b.w) exhibited almost normal renal tissues architecture with moderated leucocytes infiltration (Figure 5D), absence of glomerulus hypertrophy and degenerative changes on tubules and nephrons (Figure 5C and Figure 5E).

### 3.6 HKG restores liver and kidney antioxidant status and inhibits lipid peroxidation

Evaluation of oxidative stress marker in the liver homogenates showed that SOD and CAT activities, as well as GSH content, were significantly (*P* ˂ 0.05) reduced in heavy metals intoxicated rats when compared to control rats. Also, the MDA level, and end product of lipid peroxidation, was significantly (p ˂ 0.05) increased in liver homogenates of heavy metals intoxicated rats (Table 3). Likewise, in comparison with control rats, kidney homogenates of heavy metals-intoxicated and non-treated rats displayed a significant (*P* ˂ 0.05) decrease in GSH content, SOD, and CAT activities, and an increase in MDA level (Table 4). In contrast, HKG (25 or 100 mg/kg b.w) or silymarin (100 mg/kg b.w) daily co-treatment significantly (*P* ˂ 0.05) rescued both hepatic and renal GSH content, and SOD and CAT activities, and inhibited the elevation of MDA level, when compared to heavy metals-intoxicated rats (Table 3 and Table 4).

**Table 3:** Oxidative stress parameters in liver homogenates of rats co-treated with heavy metals mixture and HKG.

Treatments	Liver oxidative stress parameters			
	MDA (nmoL/mg protein)	GSH (nmoL/mg protein)	SOD (Unit/min/mg protein)	CAT (Unit/min/mg protein)
**Control**	7.75 ± 1.17	24.52 ± 2.41	75.46 ± 3.58	21.16 ± 1.32
**Heavy metals**	22.42 ± 2.38^**Δ**^	14.05 ± 2.19^**Δ**^	43.65 ± 2.87^**Δ**^	7.52 ± 1.43^**Δ**^
**Heavy metals + Sil (100 mg/kg)**	13.57 ± 0.97**∗**	20.09 ± 1.88**∗**	64.18 ± 3.44**∗**	15.38 ± 2.63**∗**
**Heavy metals + HKG (25 mg/kg)**	15.26 ± 1.59**∗**	18.59 ± 2.37**∗**	61.56 ± 2.80**∗**	13.11 ± 1.74**∗**
**Heavy metals + HKG (100 mg/kg)**	10.85 ± 1.09**∗**	22.86 ± 2.07**∗**	70.46 ± 3.57**∗**	17.35 ± 2.09**∗**
**HKG (100 mg/kg)**	7.60 ± 1.95^ns^	25.44 ± 1.52^ns^	72.13 ± 4.01^ns^	20.14 ± 2.32^ns^

Animals were treated daily with either demineralized water (negative control group), heavy metals mixture (heavy metals intoxicated group), HKG or co-treated with heavy metals mixture and HKG or silymarin for five consecutive months. At the end of the treatment, 10% liver homogenates were prepared and used to assess the effect of HKG on the parameters of oxidative stress in the liver (MDA, GST, SOD and CAT). Values are means ± SD, n = 6; ^Δ^ values significantly different when compared to control group (P ˂ 0.05); ^ns^ values non significantly different when compared to control group (P > 0.05); ∗ values significantly different compared to heavy metals-intoxicated group (P ˂ 0.05) using ANOVA followed by Bonferroni's post-test. MDA: Malondialdehyde; GST: Reduced Glutathione; SOD: Superoxide dismutase; CAT: Catalase; Control: Negative control group; Heavy metals: Heavy metals-intoxicated group. Sil: Silymarin; HKG: Hydro-Ethanolic (35:65, v/v) extract of *K. grandifoliola*.

**Table 4:** Oxidative stress parameters in kidneys homogenates of rats co-treated with heavy metals mixture and HKG.

Treatments	Kidney oxidative stress parameters			
	MDA (nmoL/mg protein)	GSH (nmoL/mg protein)	SOD (Unit/min/mg protein)	CAT (Unit/min/mg protein)
**Control**	5.43 ± 0.85	23.05 ± 1.89	58.24 ± 2.53	18.92 ± 1.44
**Heavy metals**	18.59 ± 0.67^**Δ**^	14.36 ± 1.17^**Δ**^	30.19 ± 3.17^**Δ**^	9.69 ± 0.81^**Δ**^
**Heavy metals + Sil (100 mg/kg)**	12.51 ± 1.08**∗**	21.51 ± 2.03**∗**	44.59 ± 1.78**∗**	16.03 ± 2.37**∗**
**Heavy metals + HKG (25 mg/kg)**	11.62 ± 1.33**∗**	18.56 ± 1.67**∗**	43.97 ± 2.18**∗**	14.74 ± 1.88**∗**
**Heavy metals + HKG (100 mg/kg)**	7.08 ± 1.13**∗**	20.49 ± 0.87**∗**	55.60 ± 1.76**∗**	16.91 ± 2.05**∗**
**HKG (100 mg/kg)**	5.66 ± 0.84^ns^	21.71 ± 2.15^ns^	60.39 ± 2.24^ns^	19.14 ± 2.16^ns^

Animals were treated daily with either demineralized water (negative control group), heavy metals mixture (heavy metals intoxicated group), HKG or co-treated with heavy metals mixture and HKG or silymarin for five consecutive months. At the end of the treatment, 10% kidneys homogenates were prepared and used to assess the effect of HKG on the parameters of oxidative stress in the kidneys (MDA, GST, SOD and CAT). Values are means ± SD, n = 6; ^Δ^ values significantly different when compared to control group (P ˂ 0.05); ^ns^ values non significantly different when compared to control group (P > 0.05); ∗ values significantly different compared to heavy metals-intoxicated group (P ˂ 0.05) using ANOVA followed by Bonferroni's post-test. MDA: Malondialdehyde; GST: Reduced Glutathione; SOD: Superoxide dismutase; CAT: Catalase; Control: Negative control group; Heavy metals: Heavy metals-intoxicated group. Sil: Silymarin; HKG: Hydro-Ethanolic (35:65, v/v) extract of *K. grandifoliola*.

### 3.7 HKG attenuates cadmium and lead accumulation in the liver and kidneys

**Figure 6:**
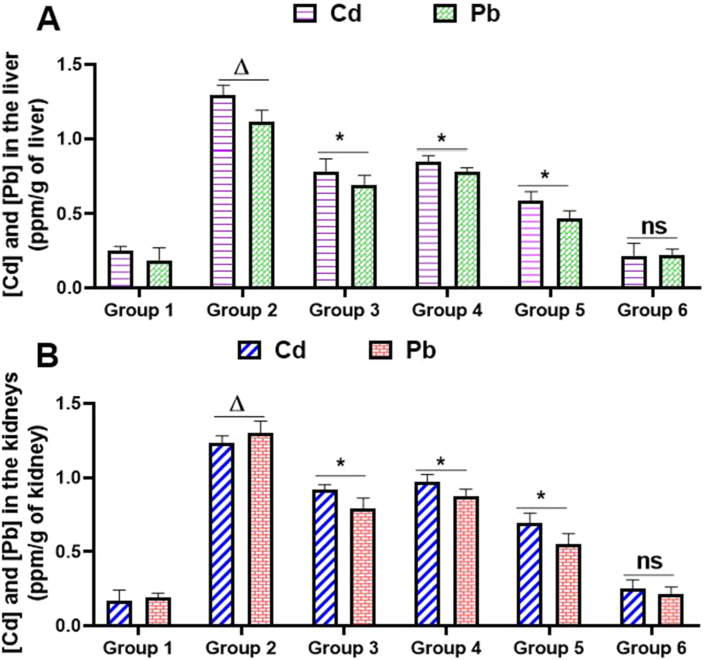
Cadmium and lead contents in the liver and kidneys of rats exposed to heavy metal mixture and treated with HKG. Animals were treated daily with either demineralized water (negative control group), heavy metals mixture (heavy metals intoxicated group), HKG or co-treated with heavy metals mixture and HKG or silymarin for five consecutive months. At the end of the treatment, 1 g of liver or kidney was mineralized and metal contents were determined by Atomic Absorption Spectrophotometry. (A) Cadmium and lead contents in the liver. (B) Cadmium and lead contents in the kidneys. Values are means ± SD, n = 6; ^Δ^ values significantly different when compared to control group (P ˂ 0.05); ^ns^ values non significantly different when compared to control group (P > 0.05); ∗ values significantly different compared to heavy metals-intoxicated group (P ˂ 0.05) using ANOVA followed by Bonferroni's post-test. Cd: cadmium; Pb: lead; Group 1: Negative control group; Group 2: Heavy metals-intoxicated group. Group 3: Heavy metals-intoxicated + Silymarin (100 mg/kg/bw/day) treated group; Group 4: Heavy metals-intoxicated + HKG (25 mg/kg/bw/day) treated group; Group 5: Heavy metals-intoxicated + HKG (100 mg/kg/bw/day) treated group; Group 6: HKG (100 mg/kg/bw/day); HKG: Hydro-Ethanolic (35:65, v/v) extract of *K. grandifoliola*.

The effects of HKG on liver and kidneys content of Cd and Pb are presented in Figure 6. As compared to normal control rats, hepatic and renal content of Cd and Pb were increased by up to 7-fold in heavy metal intoxicated and non-treated rats. However, daily co-treatment of rats with HKG (25 or 100 mg/kg b.w) or silymarin (100 mg/kg b.w) significantly (*P* ˂ 0.05) reduced Cd and Pb content in both liver and kidney, when compared to heavy metal intoxicated rats (Figure 6A and Figure 6B).

## 4 Discussion

Consumption of contaminated water seems to be one of the major routes of heavy metals contamination as reported by Angèle N. Tchana et al. (2018) who found that drinking water consumed in the city of Yaoundé, the political capital of Cameroon, contains high content of Pb, Cd, and Hg (0.9, 0.58, and 1.13 ppm respectively). Thus, taking into account that regular consumption of such drinking water may damage some vital organs such as the liver and kidneys, and therefore constitute a health risk factor for the residents, the present study aimed at assessing the potential toxicity of chronic consumption of drinking water containing high contents of Pb, Cd, and Hg on the liver and kidneys, using rats as an animal model. In addition, due to the paucity of knowledge on the protective effect of *K. grandifoliola* on heavy metal toxicity, a medicinal plant bearing hepatoprotective properties, we also evaluated the protective effect of the hydro-ethanolic stem bark extract of *K. grandifoliola* (HKG) against multi-heavy metals induced hepato-renal injuries. Aside, the chemical antioxidant properties of HKG were also investigated.

The harmful effects of heavy metal poisoning on the liver and kidneys have been well reported previously (Jaishankar et al., 2014; Mohamed et al., 2016). On one hand, heavy metals hepatotoxicity is evidenced experimentally by an elevated level of hepatic cytosolic enzymes such as alanine aminotransferase (ALT), aspartate aminotransferase (AST), and alkaline phosphatase (ALP) (Abdel-Monem et al., 2013; Balali-Mood et al., 2021). On the other hand, creatinine, a nitrogenous end product of metabolism, resulting from the breakdown of creatine and phosphocreatine, is widely accepted as an indicator for the assessment of renal function (Palipoch et al., 2022; Price and Finney, 2000). In the present study, chronic administration of Pb, Cd, and Hg at the respective concentration of 0.9, 0.58, and 1.13 ppm during five consecutive months resulted in an abnormal elevation of serum levels of ALT, AST, ALP, and creatinine from the third month to the fifth month (Figure 3). These observations correlate with severe histopathological injuries seen in liver and kidney tissues of heavy metals intoxicated and non-treated rats, characterized by the impairment of hepatic lobule architecture, severe hepatocytes necrosis and massive leucocytes infiltration (Figure 4B), and glomerulus hypertrophy, nephron and tubules necrosis (Figure 5B). However, concomitant administration of HKG (25 or 100 mg/kg b.w) or silymarin (100 mg/kg b.w) significantly (*P* ˂ 0.05) suppressed heavy metals-induced increase level of liver and kidneys function biomarkers (Table 2). These results suggest that HKG could prevent heavy metals from inducing damage to the hepatic cell membrane and renal dysfunction. In support of these biochemical outcomes, the histopathological analysis showed a decrease or complete abrogation of liver and kidneys tissues injuries by the co-treatment of rats with HKG (Figures 4 and 5). Beside these observations, we noted that administration of HKG extract alone, at the dose of 100 mg/kg b.w during the entire period of treatment did not significantly (*P* > 0.05) affect the evolution of body weight gain, as compared to non-intoxicated rats (supplementary file, Figure S1). No signs of hepatic or renal toxicity were observed, as revealed by the measurement of liver and kidneys functions biomarkers (Table 2), and the histopathological analysis (Figure 4 and 5). These results suggest that the hydro-ethanolic extract of *K. grandifoliola*, at the dose of 100 mg/kg b.w is relatively safe for the animals; and corroborate previous findings which reported the relative safeness of the aqueous extract of stem bark extract of *K. grandifoliola* at the dose of 500 mg/kg b.w in a sub-chronic toxicity study (Essama et al., 2020).

The mechanism of heavy metals toxicity is highly associated with the induction of oxidative stress which is further reinforced by their ability to accumulate in some important organs such as the liver and kidneys (Bortey-Sam et al., 2015; El-Moselhy et al., 2014). Indeed, poisoning with heavy metals goes with an excessive generation of reactive oxygen species (ROS) like NO°, HO°, and O2°. Subsequently, DNA damage, protein oxidation and lipid membrane peroxidation resulting from overproduction of ROS following heavy metals intoxication can cause pathological alteration of cell functioning, destabilization of cell membrane integrity, and cell necrosis (Balali-Mood et al., 2021; Jaishankar et al., 2014). To face against negative effects of toxic substances like heavy metals and the resulted overproduction of ROS, cells possess an endogenous antioxidant defense system consisting of antioxidant enzymes kike superoxide dismutase (SOD) and catalase (CAT), antioxidant molecules such as glutathione (GSH). This antioxidant defense system is responsible for preventing or scavenging ROS, inhibiting the oxidation of polyunsaturated fatty acids, and subsequently protects cells from oxidative stress injury (Bataille and Manautou, 2012; Jaiswal, 2004; Niture et al., 2010). Unfortunately, this antioxidant defense system is often inactivated under excessive and sustained oxidant stress conditions (Mates et al., 1999). Oxidative stress and Bioaccumulation being two important phenomena in heavy metals toxicity, we evaluated the capacity of HKG to interfere with these processes by measuring in both kidney and liver rats homogenate the level of SOD and CAT activities, GSH and MDA contents, and the concentration of Pb and Cd in liver and kidneys tissues after heavy metals intoxication. We observed in both liver and kidneys homogenates of rats receiving only heavy metals mixture, a significant (*P* ˂ 0.05) production of MDA, and a significant (p ˂ 0.05) decrease in SOD and CAT activities, as well as GSH content, as compared to the normal control rats (Table 3 and Table 4). Also, Pb and Cd in liver and kidney tissues of intoxicated and non-treated rats were approximately 7-fold higher than their respective concentrations found in hepatic and renal tissues of normal control rats (Figure 6). These findings indicate sustained oxidative stress conditions which may be associated with the excessive accumulation of heavy metals in these organs. However, simultaneous administration of HKG (25 or 100 mg/kg b.w) or silymarin (100 mg/kg b.w) significantly (*P* ˂ 0.05) suppressed the accumulation of Pb and Cd in both organ tissues (Figure 6), and heavy metals-induced overproduction of MDA, the decrease of SOD and CAT activities, and GSH content in both organ homogenates (Table 3 and Table 4). These results suggest that HKG may contribute to the excretion of heavy metals, and therefore reduce the bioaccumulation of Cd and Pb in the liver and kidneys. Also, due to its ability to restore CAT and SOD activities in heavy metals intoxicated rats, it can be suggested that HKG may up-regulate the endogenous antioxidant enzymes defense system. Indeed, these observations correlate with our previous findings demonstrating that the methylene chloride/methanol (50:50, v/v) extract of *K grandifoliola* and its isolated compounds effectively inhibit oxidative stress injury induced by H_2_O_2_, acetaminophen, and cisplatin through induction of nuclear translocation of Nrf2 (Nuclear factor-erythroid 2-related factor-2), the transcription factor responsible for the regulation of the gene expression of antioxidant enzymes (Fondjo Kouam et al., 2019; Kouam et al., 2017; Njayou et al., 2015).

The implications of free radicals and oxidant stress in heavy metals-induced hepatotoxicity and nephrotoxicity as abovementioned suggest that plant extracts and their active ingredients possessing free radical scavenging and antioxidant activities may serve as promising candidates to develop phytochemical antidotes against heavy metals toxicity. Indeed, medicinal plants bearing antioxidant activities have been shown to protect the liver and kidney against heavy metal toxicity (Amadi et al., 2019). Accordingly, we assessed the antioxidant properties of HKG to investigate whether its protective effects observed against heavy metal-induced hepato-renal toxicity could be supported by its antioxidant activities. Hence, five end-point antioxidant assays in cell-free system, including *in vitro* inhibition of lipid peroxidation; hydroxyl (HO°) and 2,2-Diphenyl-Picryl-Hydrazyl (DPPH) free radical scavenging assays; and ferric and phosphomolydenum reducing antioxidant power were used to analyze the antioxidant properties of HKG, in comparison to ascorbic acid (ASC), considered as a reference antioxidant molecule. Overall, HKG and ASC exhibited antioxidant effects with IC_50_/EC_50_ less than 25 μg/mL (Figure 2). Interestingly, for each chemical antioxidant assay, there was no significant (p > 0.05) difference between IC_50_/EC_50_ of HKG and ASC, suggesting that HKG possesses strong antioxidant properties, comparable to that of ASC. Moreover, a strong and positive correlation was observed between the phenolic compounds content of HKG and the corresponding chemical antioxidant activity. Excessive generation of ROS and lipid peroxidation being considered as critical events in heavy metals-cell death mechanism (Balali-Mood et al., 2021; Bortey-Sam et al., 2015; El-Moselhy et al., 2014; Jaishankar et al., 2014), it can be suggested that the antioxidant properties displayed by HKG, may contribute to its protective action against heavy metals-induced hepato-renal injuries in rats.

## 5 Conclusion

In the present study, we evaluated the potential toxicity of chronic consumption of drinking water containing high contents of Pb, Cd, and Hg at the respective concentrations of 0.9, 0.58, and 1.13 ppm on the liver and kidneys, and assessed the protective effect of the hydro-ethanolic stem bark extract of *K. grandifoliola* (HKG) against multi-heavy metals induced hepato-renal injuries. On one hand, our results revealed that at the tested concentrations, the solution of heavy metals mixture induces hepatic and renal damage in rats. On other hand, our data indicated that HKG exhibits hepatic and renal protection, comparable to that of silymarin, against heavy metals-induced hepato-renal injuries in rats by inhibiting oxidative stress and metals-bioaccumulation; and displays strong antioxidant activities, similar to ascorbic acid. Although in-depth studies are necessary to clearly understand the molecular mechanism underlying the protective effect of HKG on heavy metals-induced hepatotoxicity and nephrotoxicity, we recommend that drinking water supplied in developing nations should be free from toxic heavy metals contamination.

## Declarations

### Author contribution statement

Arnaud FONDJO KOUAM: Conceived and designed the experiments; Performed the experiments; Analyzed and interpreted the data; Contributed reagents, materials, analysis tools or data; Wrote the paper.

Micheline Masso; Rodrigue Fifen: Performed the experiments; Analyzed and interpreted the data; Wrote the paper.

Ferdinand Elombo Kouoh: Performed the experiments; Analyzed and interpreted the data; Contributed reagents, materials, analysis tools or data; Wrote the paper.

Ibrahim Njingou: Performed the experiments.

Angèle Nkouatchoua Tchana: Performed the experiments; Contributed reagents, materials, analysis tools or data.

Frédéric Nico Njayou: Conceived and designed the experiments; Wrote the paper.

Paul Fewou Moundipa: Conceived and designed the experiments; Contributed reagents, materials, analysis tools or data; Wrote the paper.

### Funding statement

This research did not receive any specific grant from funding agencies in the public, commercial, or not-for-profit sectors.

### Data availability statement

Data included in article/supp. material/referenced in article.

### Declaration of interest’s statement

The authors declare no conflict of interest.

### Additional information

Supplementary content related to this article has been published online at [URL].
