# Supplementary material for: Hydro-ethanolic extract of Khaya grandifoliola attenuates heavy metals-induced hepato-renal injury in rats by reducing oxidative stress and metals-bioaccumulation
Source: Heliyon. 2022 Nov 18;8(11):e11685. doi: 10.1016/j.heliyon.2022.e11685 (PMC9679385; doi:10.1016/j.heliyon.2022.e11685)
Supplement: Supplementary files [file mmc1.docx]

**Supplementary files**

**Manuscript title: Hydro-Ethanolic Extract of *Khaya grandifoliola* Attenuates Heavy Metals-Induced Hepato-Renal Injury in Rats by Reducing Oxidative Stress and Metals-Bioaccumulation**

**Authors: Arnaud Fondjo Kouam^a, b*^, Micheline Masso^b^, Ferdinand Elombo Kouoh^b^, Rodrigue Fifen^c^, Ibrahim Njingou^b^, Angèle Nkouatchoua Tchana^b^, Frédéric Nico Njayou^b*^, Paul Fewou Moundipa^b^**


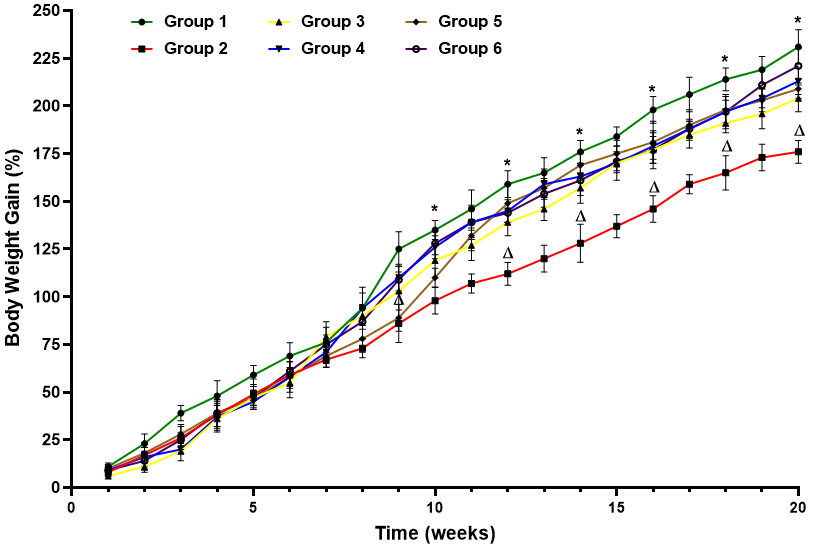


**Figure S1: Evolution of body weight gain of rats exposed to heavy metal mixture and treated with HKG**

Animals were treated daily with either demineralized water (negative control group), heavy metals mixture (heavy metals intoxicated group), HKG or co-treated with heavy metals mixture and HKG or silymarin for five consecutive months. At the end of each week, body weight of each animal was recorded and the percentage of weight gain was determined. Values are means ± SD, n = 6; ^Δ^ values significantly different when compared to control group (P˂0.05); ^*^ values significantly different compared to heavy metals-intoxicated group (P˂0.05) using ANOVA followed by Bonferroni’s post-test. Group 1: Negative control group; Group 2: Heavy metals-intoxicated group. Group 3: Heavy metals-intoxicated + Silymarin (100 mg/Kg/bw/day) treated group; Group 4: Heavy metals-intoxicated + HKG (25 mg/Kg/bw/day) treated group; Group 5: Heavy metals-intoxicated + HKG (100 mg/Kg/bw/day) treated group; Group 6: HKG (100 mg/Kg/bw/day); HKG: Hydro-Ethanolic (35:65, v/v) extract of *K. grandifoliola*.
